# Stepped-Wedge Cluster Randomised Trial of Social Prescribing of Forest Therapy for Quality of Life and Biopsychosocial Wellbeing in Community-Living Australian Adults with Mental Illness: Protocol

**DOI:** 10.3390/ijerph17239076

**Published:** 2020-12-04

**Authors:** Tamsin Thomas, James Baker, Debbie Massey, Daniel D’Appio, Christina Aggar

**Affiliations:** 1 School of Health and Human Sciences, Southern Cross University, Southern Cross Drive, Bilinga, QLD 4225, Australia; deb.massey@scu.edu.au (D.M.); christina.aggar@scu.edu.au (C.A.); 2 Primary and Community Care Services, 7/1 Central Ave, Thornleigh, NSW 2120, Australia; jbaker@pccs.org.au (J.B.); ddappio@pccs.org.au (D.D.); 3 Northern NSW Local Health District, Crawford House, Hunter Street, Lismore, NSW 2480, Australia

**Keywords:** mental disorders, loneliness, social isolation, quality of life, community services, social support, nature therapy, mindfulness

## Abstract

Social Prescribing (SP) involves linking individuals with mental illness to local health and welfare services to improve quality of life (QoL) and biopsychosocial wellbeing. SP programs address psychosocial wellbeing by linking individuals to group activities. Forest Therapy (FT) is a group nature walk with prescribed activities that promote mindfulness, relaxation, and shared experience. Improvements in psychological and physical wellbeing have been demonstrated in FT, but psychosocial impacts have not been widely investigated. This study will implement an SP FT intervention and assess the impacts on QoL and biopsychosocial wellbeing. Participants will include 140 community-living adults with mental illness at Sydney/Gold Coast, Australia. A stepped-wedge cluster randomised design will be used; each participant will complete a 10-week control period followed by a 10-week FT intervention. Weekly 90-min FT sessions will be conducted in groups of 6–10 in local nature reserves. Validated tools will measure self-report QoL and biopsychosocial wellbeing pre- and post-control and intervention periods, and 5-week follow-up. Blood pressure and heart rate will be measured pre- and post-FT sessions. Hypothesised outcomes include improvements in QoL and biopsychosocial wellbeing. This study is the first to assess SP FT, and may provide evidence for a novel, scalable mental illness intervention.

## 1. Introduction

The global prevalence of mental illness and substance use disorders is 15–20%, or over one billion people [1]. Similarly, the Australian prevalence of mental illness is 20%, and approximately half of Australians experience mental illness during their lifetime [2]. In Australia, Mental illness is the most common issue managed in General Practice (GP), being discussed in 62% of visits in 2018 [3] and placing increasing pressure on GP resources [4]. The most common course of treatment for mental illness is medication [4], however this approach only targets disease-specific neurological systems [5] and is not as effective as targeting the broad range of biological, psychological, and social factors implicated in mental illness [6].

An emerging approach to providing holistic care (treating this broad range of biopsychosocial factors) for individuals with mental illness is Social Prescribing (SP). SP involves care coordination and linkage programs where individuals with mental illness are referred to local community-based services [7]; these services can be public, private, or community-run volunteer services, and can target physical health (e.g., exercise groups, medication management), psychological health (e.g., disease-specific support groups, counselling), welfare (e.g., housing, employment services), and social support (e.g., group activities, befriending services). SP programs have been extensively implemented internationally [8]. For example a core component of the NHS England health policy is developing their SP programs which aims to increase the number of Link Workers (community care coordinators) by 1000 by 2021, and for 900,000 people to be involved in SP Programs by 2024 [9]. Similarly, SP has been implemented in Scandinavia [10], New Zealand [11], and The Netherlands[12]. SP programs have demonstrated improvements in physical wellbeing including self-reported health status [13], physical activity [14], energy, and fatigue [15,16]; psychological health such as QoL [17], depression, anxiety [18], health self-efficacy [16]; social participation [18], community participation [19], and perceived social support [13]. SP programs have also demonstrated decreased burden on the health system [14,20] including, decreased hospital admissions and GP visits [21], outpatient visits and allied health appointments [20], and prescription medication use [14].

Despite a plethora of international evidence a recent review by Bickerdike and colleagues concluded that many SP studies have a high risk-of-bias and that current evidence does not provide sufficient detail to demonstrate that SP improves participant wellbeing [22]. This review included 15 SP studies and concluded they were typically small scale, lacked comparative controls, lacked sufficient follow-up, and used non-validated tools [22]. However, given the number and variety of SP programs internationally, it is unlikely the 15 studies in this review are representative of SP. Additionally, the lack of robust evidence does not mean SP is ineffective [8]. SP interventions are necessarily translational and are implemented in a huge variety of health and community settings where formal control groups, large sample sizes, and long-term follow-ups are often not feasible due to pragmatic and financial limitations.

Regardless of this somewhat mixed evidence, SP is gaining momentum in other countries, for example Canada [23], USA [24], and Australia [8,25,26]. Thus far in Australia a small number of analogous programs have been implemented, for example the ‘How-R-U’ telephone support and referral program for elderly people discharged from hospital [27], ‘Reclink’ community-referral sport programs for at-risk youth [28], NGO Caritas’ faith-based community capacity-building programs [29], Feros Aged and Disability Care Services implementing diet, exercise, and socialisation activities [30], and most recently State and Federal government-funded programs in response to COVID-19 including the Victorian ‘Community Activation and Social Isolation Initiative’ which provides social support and welfare services via local government [31] and the ‘HeadtoHelp’ hub which provides all-ages access to mental health services [32].

However, Australia’s first holistic SP programs for individuals with mental illness and disabilities have only been active since 2016, primarily implemented by NGO Primary and Community Services (PCCS) [33,34]. These ongoing programs offer care-coordination and community referral to local public, private, and community-run health and welfare services, in addition to internal and external social activities (e.g., art classes, coffee groups). Participants in these programs have demonstrated improvements in QoL and self-assessed health status [34], biopsychosocial wellbeing, reduced health service use, and frequency and confidence in participating in social activities [33].

A social activity that may be appropriate in the context of SP due to its holistic approach to wellbeing is Forest Therapy (FT) [35]. FT is a type of Nature Therapy and is based on the Japanese practice of Shinrin-Yoku or ‘Forest Bathing’, the goal of which is a mindful submersion in nature [36]. FT involves gentle walks in nature with a prescribed series of activities such as breathing exercises, exploring textures by touching items (e.g., trees, grass), drawing a ‘sound map’, or collecting natural objects to make a group artwork [36]. Mindful exposure to nature is demonstrated to decrease physiological arousal and have a ‘calming’ effect [37]. This may be because, for example, overstimulation in urbanised cities leads to cognitive overload and attention fatigue [38], and that returning to the environment for which we are adapted is restorative [39].

Time spent in nature is demonstrated to improve psychological wellbeing [40,41], boost immune function, and promote healing [35]. For example, a 2017 review [41] assessed six international studies of healthy, unwell (alcohol dependence, hypertension, COPD), and elderly adults completing short (1 h) to long (9 day) FT sessions and concluded FT can improve hypertension, cardiac and pulmonary function, immune function, inflammation, oxidative stress, and stress hormones. The same review demonstrated psychological improvements including decreases in self-report stress, depression, and anxiety [41]. A recent (2020) review [42] of the impact of FT on mental illness assessed 20 international studies of healthy and unwell (depression, alcohol dependence, chronic pain, metabolic syndrome, stroke) adults completing short (15 min) to long (4 h) FT sessions and concluded FT improves symptoms of depression, anxiety, and anger [42]

Despite this extensive evidence regarding the physical and psychological impacts of FT, there is very little extant evidence regarding the benefits of FT on psychosocial wellbeing (e.g., social capital, loneliness, etc.). A recent review of seven SP nature-based activities concluded that these can promote socially connected and physically active communities [43]; however, the results of this review are difficult to generalise to adults with mental illness as the majority of participants were children and carer-child dyads [43]. Similarly, the results may not be applicable to FT as, while some activities included analogous activities such as visits to parks or local gardens, others included white water-rafting and visits to farmers markets [43]. Similarly, Nature Therapy more broadly has shown evidence of improving psychosocial wellbeing in the context of community gardens [44]; however, these are inherently cooperation-focussed, require infrastructure, and are only accessible to a local population.

In the context of FT, a small study (*n* = 9) in Seoul, South Korea investigated an “Urban Forest Therapy” intervention in a public park [45]. Participants attended four weekly two-hour sessions in the park, and reported “emotional bonding” with each other, in addition to improvements in self-confidence, self-worth, and self-esteem [45]. However, no further information was acquired so, although possible, it is not clear if these reported changes lead to sustained improvements in psychosocial wellbeing. Beyond these tangentially related studies, there is not a large body of literature regarding psychosocial wellbeing in FT. This may, in part at least, be because FT is generally not conducted in the context of SP and thus improvements in psychosocial functioning are not targeted or assessed.

This gap in the literature is important to investigate as the goal of SP is to holistically target wellbeing, and evidence that FT in the context of SP can improve QoL and biopsychosocial wellbeing (particularly psychosocial wellbeing) could elucidate a novel mental illness intervention. Importantly, this intervention has the potential for scalability as SP FT is affordable and flexible. SP programs can be implemented by community organisations [46], do not require specialised equipment or registered healthcare workers as activity guides, and while FT was developed in Forest biomes, it is transferable to other biomes and conditions [47]. This flexibility is particularly important in Australia, where this is large health disadvantage in regional and remote areas due to limited access to services ([AIHW [48]), and a plethora of varied landscapes. Similarly, affordability and flexibility could mean SP FT is potentially applicable and scalable on an international scale.

This study will be the first to implement and evaluate a SP FT program in Australia, and one of the first internationally. It will address some of the important limitations of previous studies [22] by including an adequate sample size, control group, clear follow-up assessments, and the use of validated tools. It will fill a gap in the research by elucidating the psychosocial impacts of SP FT in an Australian sample of adults with mental illness. It will contribute to existing practice by exploring a novel intervention in the context of SP. It will also provide initial evidence for a potential nationally scalable intervention for mental illness.

### 1.1. Aims

This study aims to assess the impact of a SP FT intervention on the QoL and biopsychosocial wellbeing of community-living adults with mental illness,

### 1.2. Hypotheses

It is hypothesised that, compared to control data (initial 10-week assessment period for all participants), over the course of the FT intervention participants will show improvements in QoL, and psychosocial, psychological, and physical wellbeing.

## 2. Methods

### 2.1. Design

This study will be a multi-site longitudinal stepped-wedge cluster randomised trial [49]. Each participant will complete an initial 10-week control period (with pre and post assessments) followed by the intervention in a group (cluster), thereby ‘acting as their own control’ [49]. Participants will be allocated to clusters in the order they enrol in the study, and commencement of each cluster will occur in random order [49]; randomisation will be conducted by author J.B. using an online random permutation generator suggested by Suresh [50]. Randomisation information will be provided in writing to Link Workers at each site who will then liaise with the intervention clinician (author D.D.) to commence clusters in the assigned order. Sites will complete the intervention sequentially.

**Figure 1:**
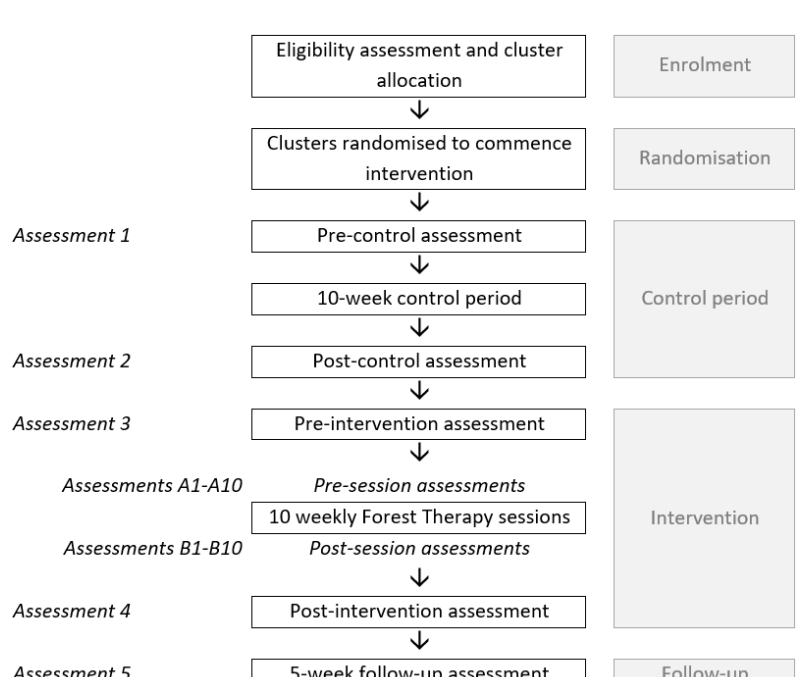
Study phases and assessment timepoints.

Assessment timepoints include pre- and post-control period (assessments 1 and 2), pre- and post-intervention (assessments 3 and 4), and 5-weeks post intervention (assessment 5). Additional timepoints include pre (assessments A1–A10) and post (assessments B1–B10) all FT sessions. A flow diagram of study phases and assessment timepoints can be seen in Figure 1, below.

### 2.2. Participants

The study aims to recruit 140 adults with ongoing mental illness (mood and psychotic disorders), living in the community at the Gold Coast (expected *n* = 45) and in Sydney (expected *n* = 95). Participants will be care-coordination clients of PCCS, an NGO that has previously developed and implemented SP programs in Australia [33,34]. PCCS has a steady flow of new care-coordination clients (who are referred by their GP) and these will form the recruitment pool. Eligibility criteria include:

#### 2.2.1. Inclusion Criteria

- Adult (aged 18+ years);
- Any sex i.e., male, female, or non-specified;
- Referred to PCCS by GP for SP program;
- Diagnosed with a mental illness (mood or psychotic disorder) *;
- Able to complete study questionnaires in English;
- Capable of providing informed consent.

* Classification of mental illnesses for inclusion are all those appearing in Section 2 of the Diagnostic and Statistical Manual of Mental Disorders, 5th Edition: DSM-5 [51], and diagnosed according. For example, neurodevelopmental disorders (autism spectrum), schizophrenia spectrum disorders, depressive and anxiety disorders, trauma-related disorders (post-traumatic stress), personality disorders, eating disorders, and substance dependence.

#### 2.2.2. Exclusion Criteria

- Receiving inpatient care in the past 6 months;
- Current thoughts of harm to self or others;
- Cognitive deficit which prevents capacity to provide informed consent or to complete study questionnaires.

## 3. Recruitment and Informed Consent

Initial assessment and recruitment of participants will be conducted by PCCS ‘Link Workers’, who are experienced social workers or registered mental health nurses. Link Workers are responsible for the initial assessment, care-coordination and linkage, and ongoing management of PCCS client wellbeing. All new clients of PCCS will be assessed for eligibility during their initial intake assessment with their PCCS Link Worker. In addition to this eligibility assessment clients will receive their usual care from PCCS involving care-coordination and community referral to local public, private, and community-run biopsychosocial health and welfare services. Informed consent will be obtained by the Link Worker; they will provide clients a FT information brochure, the Participant Information Statement (PIS), and Participant Consent Form (PCF), allow sufficient time to read both, and answer all client questions. Link Workers will emphasise that clients are not required to participate in the research project to participate in the FT program, and they may withdraw their consent at any time. Clients will indicate consent by returning a signed PCF. All clients will also receive standard care from PCCS throughout the study.

## 4. Intervention

The intervention will include 10 weekly 90-min FT sessions in groups of 6–10. Sites include Gold Coast Baree-Badalla Reserve—public nature reserve, mangrove biome, 1.2 km pathed footpath [52]; and Sydney Ku-ring-gai Wildflower Garden—‘senses tracks’, 900 m, forest biome, flat unpaved track [53]. Sessions will be facilitated by a certified FT Guide [54] and accompanying PCCS health workers (mental health nurse/allied health) with a maximum ratio of 1 staff member to 3 participants. Intervention objectives and prescribed activates follow the guidebook of the International Nature and Forest Therapy Alliance [54].

### 4.1. Intervention Objectives

The objectives of the intervention are to:

- Promote nature as a place of relaxation;
- Demonstrate mindfulness techniques;
- Develop a gratitude-based focus to enhance positive mood;
- Enhance wellbeing using a range of creative and sensory activities;
- Experience relaxing activities with others, increasing social connectedness through shared experiences;
- Develop an understanding of the importance of nature and local culture in a specific area.

### 4.2. Forest Therapy Sessions

Session structure is outlined below; an asterisk (*) indicates a session with a focus on social interactions.

1. Introduction to FT;
2. Sensory tuning activity—designed to ground participants using a combination of breathing exercises and gentle physical movement;
3. Introduction to the local environment—overview of ecological, historical, and cultural information;
4. Creative and sensory activities designed to facilitate mindfulness, including:
  a. Focusing on a specific nature point in the distance;
  b. Collecting fallen objects such as leaves and creating a collective artwork *;
  c. Creating a sound map of the area using paper/pens;
  d. Exploring textures in the natural environment by safely touching trees, grass, etc.
5. ‘Sit spot’—sit quietly and observe nature in a safe and calm environment;
6. Tea ceremony—drink tea and discuss favourite part of the experience *.

## 5. Data Collection

Assessments 1–5 will be conducted by participants’ Link Workers at PCCS offices. Additional assessments pre- and post-FT sessions will be conducted by PCCS staff at FT sites.

### 5.1. Data Matching and Confidentiality

Completed questionnaires will be non-identifiable, but for the purposes of longitudinal data-matching will include a participant-generated anonymous code based on the process of [55]. This will comprise:

- First letter of participant’s mother’s first name;
- Number of participant’s older brothers (living and deceased);
- Month participant was born;
- First letter of participant’s middle name (if none, use X).

### 5.2. Materials

#### 5.2.1. Demographics

Participant demographics will be collected at baseline (assessment 1), including age, sex, country of birth, first language, aboriginality, highest education, mental illness and disability diagnoses, employment status, living arrangements, and if they require assistance with Activities of Daily Living (ADLs).

The following tools will be used for assessments 1–5, i.e., pre- and post-control-period, pre- and post-intervention, and at 5-weeks post intervention (follow-up).

#### 5.2.2. Quality of Life

The World Health Organisation Brief assessment of QoL [56] assesses QoL over the previous 2 weeks. It is validated in adults with mental illness [57] and has good discriminant, content, and test-retest reliability [58]. Two overall items assess overall QoL and physical health, and four domains including social relationships, physical health, psychological health, and environment. Items are rated on a Likert scale from 1 (not at all/very poor) to 5 (completely/very good).

#### 5.2.3. Social Adjustment and ADLs

The Work and Social Adjustment Scale [59] assesses perceived impairment in functioning in five domains including employment, ADLs, social connection and support, and leisure activities, rated on from 0 (not at all [impaired]) to 8 (very severely [impaired]).

#### 5.2.4. Loneliness

The UCLA 3-item Loneliness Scale [60] assesses how frequently over the previous two-weeks participants have felt 1. left out, 2. isolated, and 3. lacking companionship, rated from 1 (never) to 4 (frequently).

#### 5.2.5. Depression

Patient Health Questionnaire-9 [61] assesses cognitive and behavioural depressive symptoms over the past two weeks, including mood, fatigue, appetite, and concentration, rated from 0 (not at all [experienced]) to 3 ([experienced] nearly every day).

#### 5.2.6. Anxiety

Generalised Anxiety Disorder Questionnaire [62] assesses the severity of anxiety symptoms over the past two weeks. Seven symptoms are rated according to frequency of interference with functioning from 0 (not at all) to 3 (nearly every day).

#### 5.2.7. Health Self-Efficacy

The Health Confidence Score [63] assesses health self-efficacy. Four items assess personal health knowledge, capacity to take action if needed, and perception of being included in health decisions. Items are rated from 1 (strongly agree) to 4 (strongly disagree).

#### 5.2.8. Health Service Utilisation

Health service utilisation will be assessed for the previous 4 weeks. This will include frequency of ambulance use, hospital visits, hospital admissions, and nights spent in hospital. It will also assess number of GP visits, number of allied health visits, and community health service utilisation (with details).

The following assessment tools will be used immediately prior-to and post-each FT session.

#### 5.2.9. Mood

The Profile of Mood States-Adolescents [64], validated in adults [65], assesses current mood across 6 sub-components including anger, confusion, depression, fatigue, tension, and vigour. Twenty-four items are rated based on the extent to which participants are experiencing them “right now”, rated 0 (not at all) to 4 (extremely).

#### 5.2.10. Pulse Rate and Blood Pressure

Pulse rate and blood pressure will be measured using an Omron HEM-7600T electronic upper-arm blood pressure monitor. These are designed for use by untrained lay-persons [66] but will be conducted by PCCS staff.

## 6. Outcomes of interest

### 6.1. Primary Outcomes

Global Quality of Life—WHOQoL-Bref [56]
Social Adjustment—Work and Social Adjustment Scale [59]
Loneliness UCLA 3-item Loneliness Scale [60]

### 6.2. Secondary Outcomes

#### 6.2.1. Mental Health

Depression—Patient Health Questionnaire-9 [61]
Anxiety—Generalised Anxiety Disorder Questionnaire [62]

#### 6.2.2. Health Self-efficacy

The Health Confidence Score [63]

#### 6.2.3. Physical Health

Physical Health Subscale of WHO-QoL-Bref [56]

#### 6.2.4. Health Service Utilisation

Frequency of ambulance use, hospital visits, hospital admissions, nights spent in hospital, GP visits, allied health, and community health service utilisation.

## 7. Statistical Analysis

### 7.1. Sample Size Estimation

A priori power calculation is based on self-reported quality of life WHOQoL-Bref [56] with reference values for mean and standard deviation taken from Spinal Cord Injury Research Evidence [67] and methods from Rosner [68]. Assumptions include α = 0.05, β = 0.2, and power = 0.8 (80%). Calculations indicate a sample of 126 participants is required to detect a minimum effect of 0.5 units (SD = 19.4) between control and intervention conditions post-intervention. We aimed to recruit 140 participants to allow for an attrition rate of 11%, i.e., between the 7% median attrition rate of RCTs [69] and estimated 12.9% in mental illness research [70].

#### Analysis

Data will be entered, cleaned, checked, and analysed in SPSS 27 [71]. Within and between-group analysis will be conducted with differences considered significant at α = 0.05. Analysis will be conducted according to the methods of Hussey and Hughes [72] who have produced a guide to analysis for the (relatively new) stepped-wedge cluster design. A Linear Mixed Model (LMM) will be used as this allows for non-independence in the data including clustering and repeated measures. Weighted Least Squares (Weighted Linear Regression) will be used to estimate the fixed effects. Subgroup analysis will examine differences across mental illness diagnoses. Assessment tools missing <20% responses will be imputed with participant tool mean. Tools with >20 missing responses will be excluded.

## 8. Ethical Considerations

Ethics approval for this research has been granted by the Southern Cross University Human Ethics Committee (2020/133). Protocol variations will be submitted to HREC and notification to appropriate parties conducted according to HREC recommendations. Participants in this study have a mental illness and as such are a considered a vulnerable group. All staff involved in participant contact are experienced in dealing with mental illness. Recruitment processes will ensure participants have the capacity to provide informed consent. Additionally, ongoing participant capacity to consent will be monitored by Link Workers and study staff; lack of ability to consent will result in withdrawal and referral to appropriate services. Withdrawn participants will be given the option to exclude their data from analysis.

Participants’ welfare will also be ensured throughout the study via as-usual monitoring, assessment, and service referral from PCCS Link Workers; these referrals will be participant-specific and could include referral to a range of biopsychosocial services, such as psychological and psychiatric services (including medication review) or welfare services. This frequent contact with Link Workers will also aid in retention as arising issues that limit ability to participate (for example lack of transport) fall within the purview of the Link Worker role and can be addressed. Finally, the study design has inherent ethical advantages over RCTs or crossover designs, as all participants will have access to the intervention, and the intervention will not be removed over the course of the study [49].

## 9. Compensation for Potential Bias

Potential sources of bias have been identified based on the protocol paper by Bonnici, Gerry [73] who used a stepped-wedge design.

### 9.1. Lack of Blinding and Randomisation

Given the nature of the stepped-wedge design, participants and assessment staff will not be blinded. This limitation is somewhat attenuated by the random commencement of clusters into the intervention, and the use of control data [49]. Additionally the pragmatic (controlled trial allowing for longitudinal recruitment in multiple health services) and ethical advantages of this design are sufficient to compensate for this limitation [49].

### 9.2. Heterogeneity of Participant Clusters

Participants are likely to share characteristics within study sites, and within clusters due to stepped (delayed) commencement of groups across time—particularly in the current social climate (COVID-19). This limitation is considered in the statistical approach we will use [72] which allows for non-independence in the data including clustering and repeated measures.

### 9.3. Concurrent Treatments

Over the course of the study, participants will receive ongoing assessment and referral from PCCS Link Workers which may include, for example, psychological services or medication review. This concurrent care has the potential to confound assessment of the impact of the Forest Therapy intervention. However, this regular assessment and referral will be ongoing over the course of the entire study, and thus the same for both control and intervention time periods, limiting the confounding of these treatments. Furthermore, the vulnerable nature of participants with mental illness, and the length of the study (at least 25 weeks for each participant), requires consideration of the ethics and risk of withholding other treatments. Similarly, the translational nature of this research and the study sample (vulnerable participants accessing care services in the community) limits the capacity to control a variety of variables, including access to concurrent treatments.

## 10. Discussion

This paper details the protocol for a multi-site trial of a novel intervention for Australian adults with mental illness living in the community, and under the care of a care-coordination and linkage (SP) provider. It aims to assess the impact of SP FT on QoL and biopsychosocial wellbeing. This study will capitalise on the demonstrated physical and psychological benefits of FT and implement it in the context of SP, which may also elucidate potential benefits on psychosocial outcomes. If successful, this study will provide evidence for a truly holistic biopsychosocial intervention for mental illness which has hereto not been investigated.

This study will contribute to research by addressing many of the limitations of SP studies outlined in the review by Bickerdike and colleagues [22], including using a comparatively large sample size, inclusion of follow-up assessments, a control group, and the use of validated tools. It will also use a stepped-wedge randomised trial design thereby addressing the inconstant methodological rigour of previous studies. This study will also address the limitations of previous FT interventions which have not been widely implemented in the context of mental illness, but rather general psychological distress in largely non-clinical populations—for example, healthy university students, office workers, or adults with medical conditions such hypertension [74].

This study will contribute to clinical practice as SP FT has capacity for scalability, and thus the potential to provide a, affordable, holistic intervention for mental illness in community settings on a national scale (or indeed an international scale). An important characteristic that allows scalability is that SP is not limited to care-coordination and linkage organisations; SP in general, and SP FT specifically, can be based on referred from any healthcare worker. Importantly, evidence indicates that simply ‘signposting’ community activities (such as a GP providing a flyer) is not generally effective [75], and that someone in the community that has a more involved, long-term, ‘hands on’ relationship with the participant, and facilitates access to services, is more likely be effective [46]. This may be particularly important in regional and remote areas where access to GPs is limited [76]. Additional contributors to the scalability and affordability of SP FT are the flexibility of implementation of FT which can be adapted to any biome, facilitated by non-medical-professionals, and does not require any specialised equipment [47]. This scalability is particularly important contribution to clinical practice as mental illness is ubiquitous and represents a large portion of national burden of disease and government spending, both in Australia and globally.

## 11. Conclusions

This study will implement a novel intervention for mental illness that may demonstrate improvements in QoL and across biopsychosocial domains. If successful, it will provide evidence for a novel, holistic, scalable intervention for mental illness with the potential to be delivered both nationally, and internationally.
